# Zinc homeostasis imbalance: Potential therapeutic value in neurodegenerative diseases

**DOI:** 10.4103/NRR.NRR-D-25-00632

**Published:** 2025-10-30

**Authors:** Zheyi Zhang, Wei Deng, Leilai Hu, Yulong Hu, Shenglan Zhang, Yaping Xiong, Xiao Liu, Peng Yu, Shuchun Yu, Linhui Yuan, Jing Zhang

**Affiliations:** 1 The Second Clinical Medical College, Jiangxi Medical College, Nanchang University, Nanchang, Jiangxi Province, China; 2 Department of Anesthesiology, Jiujiang University Affiliated Hospital, Jiujiang, Jiangxi Province, China; 3 Department of Anesthesiology, The Second Affiliated Hospital of Nanchang University, Nanchang, Jiangxi Province, China; 4 Department of Cardiology, Sun Yat-sen Memorial Hospital of Sun Yat-sen University, Guangzhou, Guangdong Province, China; 5 Department of Endocrinology and Metabolism, The Second Affiliated Hospital of Nanchang University, Nanchang, Jiangxi Province, China

**Keywords:** Alzheimer’s disease, neuroinflammation, neuronal cell death, oxidative stress, Parkinson’s disease, Schizophrenia, synaptic dysfunction, zinc chelators, zinc homeostasis genes, zinc signaling pathways

## Abstract

Zinc homeostasis genes are a general term for a family of genes responsible for regulating the concentration of intracellular and extracellular zinc ions, including the SLC39 (ZIP) family, the SLC30 (ZnT) family, and the metallothionein family. As an essential trace element, zinc is involved in biomolecular synthesis, energy metabolism, redox regulation, and gene expression. Recent studies have shown that abnormal expression of zinc homeostasis genes mediates neuronal apoptosis through multiple pathways, including oxidative stress and neuroinflammation. Imbalance in zinc homeostasis can result in the pathological development of various neurodegenerative disorders, including the deposition of amyloid-β in Alzheimer’s disease and the aberrant aggregation of α-synuclein in Parkinson’s disease. Therefore, regulating the expression of zinc homeostasis genes to restore normal zinc levels in vivo may be an effective strategy for treating neurodegenerative diseases. This review comprehensively summarizes the current status of research exploring zinc homeostasis genes across various family subtypes, as well as the altered expression of these genes in different neurodegenerative diseases and the underlying mechanisms. Finally, we propose zinc chelator supplementation as a novel interventional therapy for neurodegenerative diseases. This proposal includes an evaluation of the feasibility, safety, and limitations of this treatment, providing an innovative perspective for the clinical management of neurodegenerative diseases in the future.

**Facts**

• Multiple zinc homeostasis genes are abnormally expressed in neurodegenerative diseases and are closely related to oxidative stress, synaptic damage, and neuroinflammation.

• Zinc chelators and zinc supplementation therapies can bidirectionally regulate zinc homeostasis, partially reversing protein aggregation and functional impairment.

• Interdisciplinary research on the role of zinc signaling in cell death and metabolic dysregulation has accelerated the understanding of targeted drug development.

**Open questions**

• Do the changes in zinc homeostasis genes vary specifically among different neurodegenerative diseases, and can they serve as early diagnostic biomarkers?

• How can the long-term safety of zinc interventions and the optimal timing for treatment be determined?

• How can precise interventions targeting zinc homeostasis genes be achieved while effectively crossing the blood–brain barrier?

## Introduction

**Figure 1:**
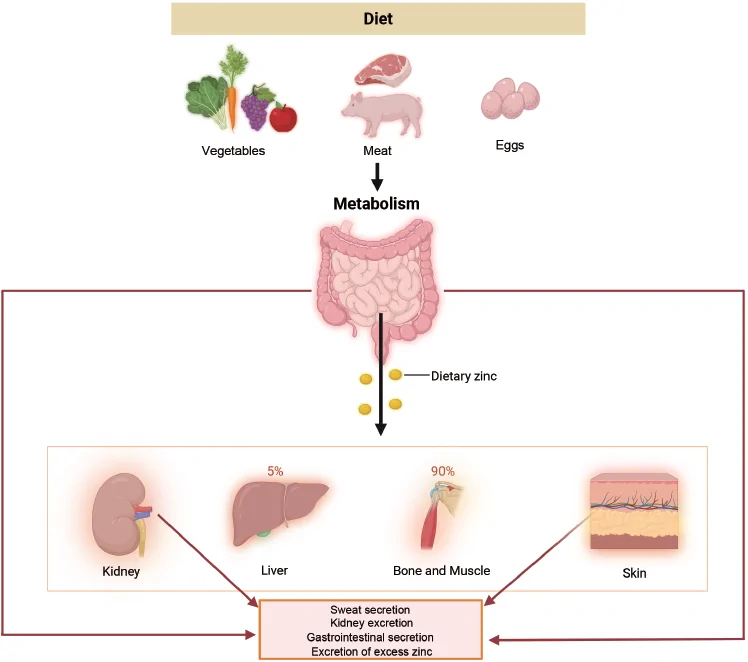
Distribution of dietary zinc in the body. Upon ingestion, digestion, and absorption, 90% of zinc is stored in the bones and muscles, while 5% is stored in the liver. The remaining zinc is distributed in various tissues and organs, such as the skin and kidneys. When zinc concentrations exceed normal levels, the body excretes excess zinc through sweat, kidney excretion, and gastrointestinal secretions. Created with BioRender.com.

Zinc, recognized as the second most important trace element essential for maintaining biological life, plays a remarkable biological role in the human body. It is involved in various cellular metabolic processes, such as DNA synthesis, gene transcription, and cell proliferation (Saper and Rash, 2009; Stiles et al., 2024). Similar to other essential trace elements, zinc is a component of most proteins in the body. Approximately one-tenth of the proteins in the body are classified as zinc proteins; zinc influences the functions of various enzymes and transcription factors, suggesting its significance at the physiological level (Maywald and Rink, 2024). Chen et al. (2024) have shown that the adult human body contains 2–3 g of zinc, with 90% stored in the bones and muscles, about 5% in the skin and liver, and the remaining 2%–3% in other tissues and organs. The retina, prostate, kidneys, and liver have considerably higher concentrations of zinc than other organs (**Figure 1**).

Zinc homeostasis genes are categorized into three families: the SLC39 (ZIP) family, the SLC30 (ZnT) family, and the metallothionein (MT) family. ZIP encodes zinc transport proteins primarily responsible for the inward transport of zinc, such as transferring Zn^2+^ from the extracellular environment or intracellular vesicles to the cytoplasm (Liu et al., 2024). In contrast, ZnT-encoded zinc transporter proteins are mainly responsible for transferring Zn^2+^ from the cytoplasm to the extracellular environment or intracellular vesicles. This complementary relationship helps maintain intracellular zinc levels in a relatively stable state (Chen et al., 2024). The elevated sulfhydryl levels of MTs confer remarkable antioxidant properties, enhancing their ability to scavenge free radicals and mitigate oxidative stress damage. Consequently, alterations in MT expression are a common mechanism of oxidative stress in humans (Yang et al., 2024b). The incidence of neurodegenerative diseases is escalating annually, indicating a significant gap in research exploring their treatment. A recent study conducted by Wilson et al. (2023) summarizes eight hallmarks of neurodegenerative diseases: pathological protein aggregation, synaptic and neuronal network dysfunction, abnormal protein homeostasis, cytoskeletal abnormalities, altered energy homeostasis, DNA and RNA defects, inflammation, and neuronal cell death. As research on neurodegenerative diseases progresses, advances have been made in understanding the pathogenesis of some conditions. For example, the deposition of amyloid-β (Aβ) protein in Alzheimer’s disease (AD) is thought to play a vital role in the pathological mechanisms underlying the disease (Zhang et al., 2023). In Parkinson’s disease (PD), abnormal aggregation of α-synuclein and widespread dispersion of pathology between the brainstem, gut, and higher brain regions are central to its development (Morris et al., 2024). Recent studies also suggest that immune and inflammatory responses in the gut that trigger maladaptation accelerate PD pathogenesis (Morris et al., 2024; Roodveldt et al., 2024). However, the pathogenesis of some neurodegenerative diseases remains controversial and requires further comprehensive research. For example, some researchers contend that the aberrant amplification of CAG repeats in the Huntington gene, leading to the formation of aggregates of Huntington’s protein in neuronal nuclei and synapses, is an important mechanism in Huntington’s disease (HD) (Tong et al., 2024). Additionally, it has been proposed that mitochondrial toxins such as 3-nitropropionic acid may play a role in the pathogenesis of HD by inducing the selective death of medium spiny neurons in the striatum (Tabrizi et al., 2020). Manoli and State (2021) demonstrate a significant accumulation of autism spectrum disorder (ASD) risk on autosomes; however, this does not adequately account for the increased prevalence of harmful mutations in females compared with males in ASD (Manoli and State, 2021). It has been hypothesized that this phenomenon may be related to gender variation in the brain. ASD pathogenesis expresses itself in different brain regions and overlaps based on remote connections (Manoli and Tollkuhn, 2018). However, this speculation requires further confirmation in future research.

Previous studies have revealed a substantial association between alterations in zinc homeostasis genes and the development of neurodegenerative diseases (Lee et al., 2022; Wang et al., 2023). Zinc homeostasis genes play a considerable role in the pathogenesis of neurodegenerative diseases through mechanisms such as oxidative stress, neuroinflammation, synaptic dysfunction, and zinc-mediated neuronal cell death. Therefore, targeting the expression of zinc homeostatic genes to alleviate these alterations may be a new strategy for treating neurodegenerative diseases. However, there remains a lack of specific signaling pathways and molecular insights into zinc homeostatic genes in the pathology of neurodegenerative diseases, which may contribute to the insufficient clinical translation of zinc homeostatic gene modulation therapies.

In this review, we have examined the current status of research on zinc homeostasis genes and summarized the alterations in these genes across different subtypes of neurodegenerative diseases. We have also discussed the potential value of zinc homeostasis genes in treating neurodegenerative diseases and evaluated the safety and feasibility of this treatment to establish a theoretical foundation for the clinical translation of zinc homeostasis gene therapy, which may offer new hope for the clinical treatment of neurodegenerative diseases.

## Search Strategy

Studies included in this review were extracted from the PubMed database, with the majority published between May 2018 and September 2024. The following MeSH terms were used to search for relevant literature: “zinc homeostasis genes,” “Alzheimer’s disease,” “Parkinson’s disease,” “schizophrenia,” “synaptic dysfunction,” “zinc transporter protein,” “neurodegenerative diseases,” and “zinc therapy.” This review only involved studies investigating the relationship between zinc homeostasis genes and neurodegenerative diseases. Our analysis focused on the role of zinc homeostasis genes in neurodegenerative diseases and their potential therapeutic value.

## Zinc and Neurodegenerative Diseases

### Zinc in the nervous system

Zinc is relatively predominant in the brain, with an estimated concentration of 150 μmol/L, which is approximately 10 times higher than serum zinc concentrations. Approximately 70% of zinc in the brain constitutes a vital component of protein structure, maintaining the normal activity of about 2000 transcription factors and more than 300 enzymes that facilitate various biological processes in the body. Studies have shown that 10%–15% of zinc exists in a “free” or “bioavailable” form, which is highly concentrated in the synaptic vesicles of a subpopulation of glutamatergic neurons (Chen et al., 2024). Ashraf et al. (2019) discovered an uneven, distinctive distribution of zinc in the brain, with notable concentrations predominantly identified in specific regions. The highest concentrations of zinc were observed in the hippocampus, followed by the cortex and striatum, while the lowest concentrations were found in the cerebellum. They also identified a positive correlation between the density of neuroglia (glial cells) in the hippocampus and cortex and the zinc concentration in this neuroglia, suggesting that neuroglia play an integral role in regulating zinc levels in the brain). Rychlik et al. (2022) found that defective expression of the zinc-sensing receptor (GPR39) leads to deficiencies in situational memory and spatial memory, and also affects the expression of age-related genes in the hippocampus of mice. Imbalances in zinc homeostasis in the brain have been associated with numerous disorders of the central nervous system (CNS). A significant mechanism underlying AD development is the accumulation of Aβ. Increased levels of zinc during synaptic transmission contribute to the deposition of Aβ, suggesting that zinc plays a crucial role in the pathogenesis of AD (Ossenkoppele et al., 2022). Both zinc deficiency and excess are associated with the development of PD. Moderate amounts of zinc inhibit 6-hydroxydopamine-induced oxidative stress and reduce dopaminergic neurotoxicity induced by methamphetamine by increasing the expression of MTs, thereby preventing the production of reactive oxygen species and ultimately reducing the onset of PD (Sikora and Ouagazzal, 2021). However, excessive zinc levels increase oxidative stress by activating nicotinamide adenine dinucleotide phosphate oxidase and depleting glutathione (GSH), leading to cytotoxicity, neuronal loss, and accelerated progression of PD (Sikora and Ouagazzal, 2021). We have presented a brief overview of the general functions of zinc in the human body and investigated its link to neurological functions, establishing a rigorous foundation for the subsequent introduction of zinc homeostasis genes.

### Zinc imbalance and neurodegenerative diseases

Maintenance of neurological function is inextricably linked to neuronal function. Zinc is involved in the genesis of neurons, crucial for maintaining neurological functions in humans. Previous studies have shown that zinc can reverse neurological impairment in aluminium-induced neurodegeneration by modulating molecular pathways mediated by α-synuclein and amyloid precursor protein (Chen et al., 2024). A study conducted in rodents found that the occurrence of zinc deficiency during pregnancy was associated with reduced cell counts and localized brain mass in the cerebellum, limbic system, and cerebral cortex (Georgieff et al., 2018). Zinc depletion resulting from early postnatal Giardia flagellosis may induce the death or dispersion of pyramidal neurons in the CA1 and CA3 regions of the hippocampus. This condition may cause thinning or loss of laminae and a significant reduction in cerebellar Purkinje cells, which ultimately impact the development of brain functions. Mutations in ZNF292, the gene encoding the highly conserved zinc finger protein, affect the normal transmission of multiple pathways, resulting in a range of neurodevelopmental abnormalities, such as intellectual disability, ASD, and attention deficit hyperactivity disorder (Alizadeh et al., 2020). Maternal exposure to di-2-ethylhexyl phthalate during pregnancy reduces zinc concentrations of fetal cortical tissue. Therefore, this reduction in zinc levels ultimately leads to the downregulation of ERK1/2 and neurodevelopmental damage by regulating the activity of the phosphatase PP2A (Liu et al., 2022).

## Zinc Homeostasis and Zinc Homeostasis Genes

### Zinc homeostasis

**Figure 2:**
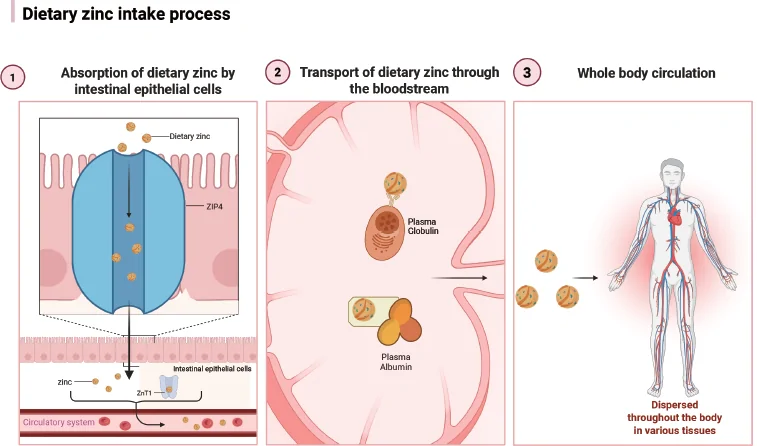
Process of zinc intake *in vivo*. During digestion, various enzymes induce the release of dietary zinc, which is then absorbed into the enterocytes via ZIP4 in the membrane of the intestinal epithelial wall. Subsequently, with the assistance of ZnT1, dietary zinc enters the bloodstream and is transported to various systemic tissues by the combination of plasma albumin and plasma globulin and then utilized. Created with BioRender.com.

Zinc homeostasis is the body’s mechanism that regulates and maintains intracellular zinc within a constant state. The regulation of zinc homeostasis is crucial for maintaining appropriate concentrations of zinc in the body and is managed by ZnTs, zinc-regulated transport proteins (ZIPs), and MTs. ZIPs are responsible for the translocation of Zn^2+^ from the extracellular milieu or intracellular vesicles into the cytoplasm (Wiuf et al., 2022). In contrast, ZnTs translocate Zn^2+^ from the cytoplasm to the extracellular environment or intracellular vesicles. This complementary interaction maintains the homeostasis and stability of intracellular Zn^2+^ concentration (Kambe et al., 2015). MTs play an essential role in regulating the binding, release, and distribution of Zn^2+^ to maintain intracellular zinc homeostasis through redox regulation. Dietary zinc transport involves the absorption of Zn^2+^ present in the diet from the gastrointestinal tract into the cells, mediated by ZIP4 on the parietal membrane structure of the intestinal epithelial cell. Subsequently, ZnT1 mediates zinc transfer from the intestinal epithelial cell into the blood circulation in the body. Subsequently, Zn^2+^ binds to plasma proteins such as albumin and globulin and is transported to the tissues through the blood, where it is eventually utilized for cellular activities (Aras and Aizenman, 2011). With the advancement of structural and biochemical studies on ZnT, the mechanism of transport is increasingly understood (Chen et al., 2024). Research showed that ZnT transporter proteins and their homologues are involved in the transportation of zinc ions as Zn^2+^/H^+^ reverse transporter proteins, with the primary energy supply for their transport being the concentration gradient of vesicular H^+^ (Hara et al., 2017). Since the structure of the ZIP family of transporter proteins has been proven elusive, their specific mechanisms are currently being explored. MTs, a family of cysteine-rich and low molecular weight proteins, are the most abundant zinc storage proteins in the human body. It has been shown that zinc exchange occurs within MT, and various molecules can facilitate the release of zinc from these proteins. The findings of dynamic translocation of MT within cellular compartments, which requires additional zinc, suggest that MT could be a major source of readily available zinc (Spahl et al., 2003; **Figure 2**).

In the above section, the process of zinc absorption in the body was described. The following sections will detail zinc homeostasis genes and elucidate their functions in the human body.

### Zinc homeostasis genes

#### Classifications and functions of zinc homeostasis genes

**Figure 3:**
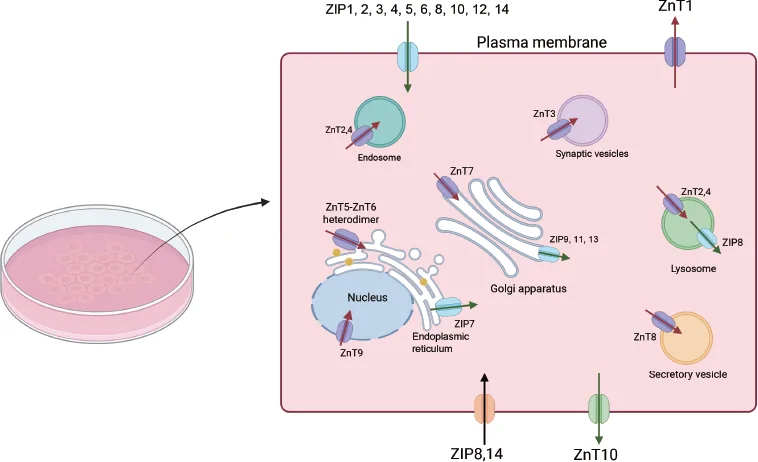
Cellular localization of zinc transporter proteins and their functions. The blue and orange transporter proteins in the figure represent the ZIP family, while the purple and green transporter proteins are ZnT family proteins. The green arrows indicate the direction of zinc transport via ZIP family proteins, whereas the red arrows show the transport direction of zinc by ZnT family proteins. Furthermore, ZIP8, ZIP14, and ZnT10 are unique zinc transporter proteins because they can facilitate the transport of both zinc and manganese (Felber et al., 2019; Steimle et al., 2022). The black arrows represent the transport directions of zinc and manganese mediated by ZIP8, ZIP14, and ZnT10. Notably, ZnT8 can be expressed in the membranes of cytoplasmic vesicles, mediating the transport of cytoplasmic zinc to secretory vesicles in pancreatic β-cells (Kasinathan et al., 2024). Additionally, ZnT5 and ZnT6 generally associate to form a ZnT5-ZnT6 heterodimer, which is responsible for mediating zinc transport in the endoplasmic reticulum (Yuasa et al., 2024). Created with BioRender.com.

Zinc homeostasis genes refer to genes that regulate the intracellular homeostasis of zinc ions, including the ZIP (*SLC39*) gene family, the ZNT (*SLC30*) gene family, and MT genes (Lyu et al., 2022). The effect of ZIP genes on the pathogenesis of cancer and diabetes has garnered substantial attention in recent research. Wan and Wang (2022) showed that the expression levels of *SLC39A1*, *SLC39A3*, *SLC39A4*, *SLC39A6*, *SLC39A7*, *SLC39A10*, and *SLC39A13* in hepatocellular carcinoma tissues were significantly higher than those in healthy tissues. However, the expression levels of *SLC39A5*, *SLC39A9*, *SLC39A11*, and *SLC39A14* were lower in tumor tissues compared with normal tissues, which has auxiliary value in the clinical diagnosis of hepatocellular carcinoma. By studying the prognosis of several patients diagnosed with hepatocellular carcinoma, they found that the overexpression of *SLC39A1*, *SLC39A6*, or *SLC39A13* is an influential factor for poor prognosis in patients with hepatocellular carcinoma. Notably, the increased expression of *SLC39A6* mainly occurred in the advanced stage of hepatocellular carcinoma, while its expression remained relatively stable in the early stage. This variation in expression levels holds considerable potential for enhancing the clinical diagnosis of hepatocellular carcinoma (Wan and Wang, 2022). Conversely, Zeng et al. (2019) showed that ZIP4 can induce epithelial-mesenchymal transition, promoting the migration and invasion of nasopharyngeal carcinoma through the PI3K/Akt signaling pathway. In a study addressing diabetes mellitus, Wang et al. (2019) demonstrated that insulin secretion was blocked and glucose tolerance in the pancreatic islets was reduced in *Slc39a5* knockout mice. The underlying mechanism involved *Slc39a5*-mediated zinc endocytosis, which induced the expression of Glut2 through Sirt1-mediated activation of Pgc-1α. This finding offers a novel perspective for the treatment of diabetes mellitus and its related complications (**Figure 3**).

#### SLC39 (ZIP) family

ZIP family proteins are a class of transmembrane proteins characterized by eight transmembrane structural domains. It is believed that these proteins form homodimers consisting of eight transmembrane helices, with two prominent histidine residues located in transmembrane helices IV and V, which are associated with the intramembrane zinc-binding sites (Kambe et al., 2015). While ZIP transporter proteins are primarily recognized for their role in zinc transport, they also facilitate the transport of other metal ions. For example, ZIPs are involved in the transport of iron, manganese, cadmium, and copper (Ahmad et al., 2023). The next section describes the relevant roles of currently known zinc transporter proteins (**Additional Table 1**).

**Additional Table 1:** Functions and localization of the ZIP family

Subtype	Tissue localization	Subcellular localization	Function	Neurodegenerative disease	Reference
ZIP1	Ubiquitous	Plasma membrane	Transport of Zn^2+^ into cells	Alzheimer's disease	Lioumi et al., 1999; Gaither and Eide, 2001; Zhao et al., 2014
ZIP2	Prostate, uterus, ovary	Plasma membrane	Transport of Zn^2+^ into cells	Autism spectrum disorder	Guo et al., 2023
ZIP3	Testicular	Plasma membrane	Transport of Zn^2+^ into cells	Not described	Dufner-Beattie et al., 2005; Kambe et al., 2015
ZIP4	Colon, duodenum, jejunum, kidneys, stomach	Plasma membrane, endosomal membrane	Transport of Zn^2+^ into cells	Autism spectrum disorder	Guo et al., 2024b
ZIP5	Epithelial cells, pancreatic alveolar cells	Plasma membrane	Transport of Zn^2+^ into cells	Not described	Dufner-Beattie et al., 2004; Gaudet et al., 2011
ZIP6	Breast, kidney, prostate, pituitary gland	Plasma membrane	Transport of Zn^2+^ into cells	Not described	Nimmanon et al., 2021; Fan et al., 2024
ZIP7	Ubiquitous	Endoplasmic reticulum, Golgi membrane	Transport of Zn^2+^ from the endoplasmic reticulum/Golgi to the cytoplasm	Not described	Taylor et al., 2004; Huang et al., 2005
ZIP8	Ubiquitous	Plasma membrane, lysosomal membrane	Transport of Zn^2+^ and Mn^2+^ into the cells Transport of Zn^2+^ from the lysosome to the cytoplasm	Not described	Zang et al., 2016; Winslow et al., 2020
ZIP9	Ubiquitous	Plasma membrane Golgi Lysosome	Transport of Zn^2+^ into the cells Transport of Zn^2+^ from the Golgi/mitochondria to the cytoplasm	Not described	Matsuura et al., 2009; Pal et al., 2014
ZIP10	Ubiquitous	Plasma membrane	Transport of Zn^2+^ into the cells	Schizophrenia	Pal et al., 2014
ZIP11	Testis, stomach, cecum, Colon	Nucleus, Golgi	Transport of Zn^2+^ into the cells	Not described	Kelleher et al., 2012; Martin et al., 2013; Olea-Flores et al., 2022
ZIP12	Ubiquitous, brain, testis	Plasma membrane	Transport of Zn^2+^ into the cells	Alzheimer's disease, autism Parkinson's disease, schizophrenia	Strong et al., 2020; Davis et al., 2021; Ye et al., 2022; Dean et al., 2024
ZIP13	Ubiquitous	Golgi and vesicle	Transport of Zn^2+^ into the cells Transport of Zn^2+^ from the Golgi to the cytoplasm	Not described	Shoji et al., 2024
ZIP14	Jejunum, liver, heart	Plasma membrane	Transport of Zn^2+^ and Mn^2+^ into the cells	Not described	McCabe and Zhao, 2024

This table summarizes the tissue and subcellular localization of the ZIP family of zinc transporter proteins, as well as the functions of the ZIP family isoforms and the types of neurodegenerative diseases associated with altered expression.

*ZIP8*

ZIP8 is expressed in a wide range of tissues and is located in the cellular and lysosomal membranes. It mediates the transport of various metal ions *in vivo*, including the cellular uptake of Zn^2+^, Mn^2+^, and Zn^2+^ transport. This protein primarily facilitates the translocation of Zn^2+^ from lysosomes to the cytoplasm (Zang et al., 2016; Winslow et al., 2020). The Schizophrenia Working Group of the Psychiatric Genomics Consortium (2014) has identified a strong association between genetic mutations in ZIP8 and schizophrenia by exploring 108 genetic loci related to the disorder. When ZIP8 mutates, it may alter the recapture of zinc, leading to increased zinc concentrations in the extracellular matrix. This increase can result in sustained inhibition of NR2A-containing N-methyl-D-aspartate (NMDA) receptors, aligning with the hypoglutamatergic hypothesis of schizophrenia (2014).

*ZIP9*

ZIP9 is expressed in various tissues and is located in the perinuclear region and at the plasma membrane, where it mediates Zn²⁺ transport from the Golgi or mitochondria to the cytoplasm (Matsuura et al., 2009; Thomas et al., 2014). Previous research conducted in mice revealed that androgens improve learning, memory, and synaptic plasticity by influencing the expression of ZIP9 (Song et al., 2023). Notably, ZIP9 mediates the effects of dihydrotestosterone through the ERK1/2-eIF4E pathway and influences the expression of hippocampal synaptic proteins, including excitatory postsynaptic density (PSD) 95, drebrin, SYP, and dendritic spine density, thereby affecting learning and memory in amyloid precursor protein (APP) and presenilin 1 (PS1) mice (Song et al., 2023). Furthermore, ZIP9 impacts B-cell signaling pathways by regulating zinc levels in the cytoplasm, leading to the phosphorylation of Akt and Erk (Taniguchi et al., 2013). The underlying mechanism involves ZIP9 mediating dihydrotestosterone through the ERK1/2-eIF4E pathway, which affects the expression of hippocampal synaptic proteins, including PSD95, drebrin, SYP, and dendritic spine density in APP/PS1 mice, ultimately influencing learning abilities and memory in these mice (Song et al., 2023).

*ZIP12*

ZIP12 is expressed in various tissues, with higher expression observed in the testis and the CNS. It is mainly localized at the plasma membrane and imports extracellular zinc into the cytoplasmic lysate (Davis et al., 2021; Ye et al., 2022). Among the entire ZIP family, ZIP12 has the highest zinc affinity (Chowanadisai et al., 2013) and is also involved in neurite growth and enhancing mitochondrial function in neuronal cells (Davis et al., 2021). Increased expression of ZIP12 mRNA in the brain regions of patients with schizophrenia suggests its potential role in the pathogenesis of CNS disorders (Davis et al., 2021). Chowanadisai et al. (2013) have shown that ZIP12 is involved in neuronal differentiation and neurite formation. The knockdown of this protein reveals its ability to reduce the activation of cAMP-responsive element-binding protein and phosphorylation at the locus of serine 133, which serves as a key pathway for neuronal differentiation. Additionally, the knockdown of ZIP12 using antisense morpholino impairs neural tube closure, prevents development during neurulation, and reduces the polymerization of microtubule proteins in the neural plate. Zhang et al. (2015) reported that rare mutations or common polymorphisms in *SLC39A12* may predispose individuals to increased susceptibility to neurodegenerative diseases. The study conducted by Zhang et al. (2015), which compared healthy subjects without mental illness to those with schizophrenia, identified the most differentially expressed genes in Brodmann’s areas (BA) 8, 9, and 44. Compared with control subjects without psychiatric disorders, certain variants of ZIP12 were highly expressed in individuals with schizophrenia in the examined regions of the brain, including the frontal lobe, superior frontal gyrus, and inferior frontal gyrus. These results revealed a strong correlation between ZIP12 and schizophrenic neuropathology in BA 8, 9, and 44 cortical regions, suggesting that ZIP12 and its ability to transport zinc may play a key role in the pathogenesis of schizophrenia. However, to the best of our knowledge, no study has confirmed whether genetic variations in *SLC39A12* contribute to the risk of autism. Polymorphisms of ZIP12 and rare variants have been correlated with structural MRI findings of gray matter volumes in the basal ganglia and the total nucleus accumbens. Additionally, differences in the volumes of the caudate and shell nucleus were detected in patients with autism, suggesting a plausible risk of autism attributed to the effects of ZIP12 in neuromorphogenesis and early neural development (Chowanadisai et al., 2013). Davis et al. (2021) demonstrated high expression of ZIP12 in the brain and correlated it with brain metal content using magnetic susceptibility-weighted MRI, indicating that ZIP12 may influence neurological and psychiatric disorders such as PD, AD, and schizophrenia.

#### SLC30 (ZnT) family

ZnT transporter proteins are located in various intracellular compartments and plasma membranes, where they function as either Zn^2+^ or H^+^ exchangers to facilitate the efflux of zinc from cytoplasmic lysates and compartmentalize it into intracellular organelles and vesicles—performing the opposite function of the ZIP protein family. The classification by Kambe et al. (2006) divides the ZnT protein family into four groups: (1) ZnT1 and ZnT10; (2) ZnT2, ZnT3, ZnT4, and ZnT8; (3) ZnT5 and ZnT7; and (4) ZnT6. However, the structures of ZnT proteins are not fully understood; it is hypothesized that these gene families have six transmembrane structural domains (TMDs), with the N- and C-terminal regions oriented towards the cytoplasmic lysate (Kambe, 2012). Unlike other proteins within this family, ZnT5 proteins have an unusually long amino-terminus that includes a region where nine putative TMDs are fused to six conserved TMDs; the function of this region is not completely elucidated and requires further study (Kambe et al., 2002). The structure of YiiP, the ZnT homologue in *E. coli*, has been experimentally determined, facilitating a thorough understanding and exploration of the structure of ZnT. YiiP forms homodimers; each monomer has six TMDs, with both the N-terminus and C-terminus located on the cytoplasmic side of the membrane. Each YiiP protomer contains four zinc binding sites. Furthermore, the site within the TMD (site A) is the major zinc-transporting site; the other site is located at the interface between the membrane and the cytoplasmic structural domains (site B), while the remaining two are situated in the cytoplasmic structural domains (site C), which have a band of abba-folded metal chaperone-like structure but lack sequence homology with known metal chaperones (Lu and Fu, 2007; Lu et al., 2009). Based on the structure of YiiP, structural models have been generated for ZnT3, ZnT5 (which presents six TMDs at the C-terminus), and ZnT8. The four hydrophilic residues in TMDs II and V form a zinc-binding site corresponding to site A in YiiP, which is highly conserved. However, aspartic acid is replaced by histidine in the ZnT transporter protein, and this site change may be relevant to zinc-transport activity (Ohana et al., 2009). Thus, the roles of ZnT family genes will be further elucidated below (**Additional Table 2**).

**Additional Table 2:** Functions and localizations of the ZnT family

Subtype	Tissue localization	Subcellular localization	Function	Neurodegenerative disease	Reference
ZnT1	Ubiquitous	Plasma membrane	Transport of Zn^2+^ out of cells	Alzheimer's disease	Gottesman et al., 2022; Stiles et al., 2024
ZnT2	Breast, colon, pancreas	Endoplasmic reticulum, endosomes, lysosomes	Transport of Zn^2+^ into endosomes, zymogen granules, mitochondria, and secretory vesicles; Transport of Zn^2+^ out of cells		Abdo et al., 2021; Kelleher et al., 2022
ZnT3	Amygdala, cerebral cortex, hippocampus	Presynaptic membrane	Transport of Zn^2+^ from the cytoplasm into synaptic vesicles	Alzheimer's disease, epilepsy, Huntington's disease, schizophrenia	Medina-Vera et al., 2023; Qi et al., 2023; Guo et al., 2024a
ZnT4	Airway epithelium, kidney, mammary gland, prostate	Endosome membrane, lysosomal membrane	Transport of Zn^2+^ from the cytoplasm into endosomes or lysosomes		Amagai et al., 2023; Ishida et al., 2025
ZnT5	Pancreas, intestine, prostate	Endoplasmic reticulum, Golgi	Transport of Zn^2+^ from the cytoplasm into organelles	Depression	Ueda et al., 2022; Amagai et al., 2023
ZnT6	Brain, liver	Golgi	Transport of Zn^2+^ from the cytosol into the organelle	Alzheimer's disease	Olesen et al., 2016; Amagai et al., 2023
ZnT7	Ubiquitous	Cytoplasmic vesicles, Golgi	Transport of Zn^2+^ from the cell membrane into the Golgi		Bui et al., 2023
ZnT8	Pancreatic beta cells, retinal pigment epithelial cells	Cell membrane, Cytoplasmic vesicle membrane	Transport of Zn^2+^ from the cytoplasm into secretory vesicles		Chimienti et al., 2006; Davidson et al., 2014
ZnT9	Liver, neuroglia, prostate	Mitochondrial membrane	Transport of Zn^2+^ from the mitochondria to the cytosol	Intellectual disability	Ma et al., 2022; Ge et al., 2024
ZnT10	Brain, liver, prostate	Endosome, Golgi membrane	Transport of Zn^2+^ and Mn^2+^ out of cells	Alzheimer's disease, Parkinson's disease	Jackson et al., 2022; McCabe and Zhao, 2024

This table summarizes the tissue and subcellular localization of the ZnT family of zinc transporter proteins, as well as the functions of the ZnT isoforms and the types of neurodegenerative diseases associated with altered expression.

*Z* *nT1*

Unlike the other proteins in the family, ZnT1 is the only ZnT transporter protein primarily localized at the plasma membrane, where it exports cytoplasmic zinc to the extracellular space. The expression of ZnT1 is regulated post-translationally by cellular zinc levels (Yang et al., 2024a). When zinc levels are sufficient, ZnT1 accumulates at the plasma membrane, supporting its role in zinc efflux. Conversely, under zinc-deficient conditions, ZnT1 molecules at the plasma membrane are endocytosed and degraded via the proteasome and lysosomal pathways (Yang et al., 2024a). Cytoplasmic zinc transport is mediated by ZnT1, directing zinc towards the extracellular space near the NMDA receptor (NMDAR), where binding to the highly zinc-sensitive NMDAR subunit GluN2A inhibits NMDAR function (Krall et al., 2020). However, the link between ZnT1 and AD is not fully elucidated; some researchers suggest that the tissue-specific function of ZnT1 may serve as a therapeutic target for AD (Schweigel-Röntgen, 2014).

*ZnT3*

ZnT3 is abundantly expressed in neuronal cells, particularly in the hippocampus, amygdala, and cerebral cortex, with levels higher than in other tissues (Qi et al., 2023). A recent study showed that WFS1 regulates the maintenance of homeostatic and apoptotic zinc (Zn^2+^) balance in neural progenitor cells and brain-like organs in the context of dysregulated lipid metabolism by inhibiting ZnT3 (Gong et al., 2024). Alterations in the expression levels of ZnT3 have been associated with the onset of several abnormalities in the CNS. Niu et al. (2020) investigated the relationship between HD and ZnT3 expression by examining the pathogenesis of HD in transgenic mice. HD proteins inhibit ZnT3 expression by repressing Sp1 binding to the ZnT3 promoter, and the downregulation of ZnT3 disrupts zinc homeostasis in synaptic vesicles, ultimately leading to early synaptic dysfunction and neurological deficits in HD. Research has indicated that the regulation of Zn^2+^ levels in the brain is linked to AD. Additionally, the accumulation of Aβ peptides within amyloid plaques is a defining pathological feature of AD. The binding of Aβ to Zn^2+^ and their interaction promotes the aggregation of Aβ on solid surfaces (Ha et al., 2007). Analysis of postmortem brains from individuals who suffered from AD revealed a significant loss of ZnT3 expression in cortical regions, suggesting that neuronal cells are particularly affected by the reduced expression of ZnT3 mRNA in the disease. Alterations in neuronal Zn^2+^ levels induced by changes in ZnT3 expression may occur early in the pathogenesis of AD (Beyer et al., 2009). Nonetheless, low concentrations of zinc mediated by ZnT3 modulate the activity of various voltage-gated and ligand-gated ion channels, which are involved in the alterations observed in many pathophysiological CNS disorders, including epilepsy, schizophrenia, and AD (Marger et al., 2014).

*ZnT5*

ZnT5 is expressed in the pancreas, intestines, and prostate. It is predominantly localized in the Golgi and the ER, with its differential subcellular localization determined by different regions in the C-terminal (Molina-López et al., 2024). It is responsible for mediating the flow of zinc from the cytoplasm to the vesicles, thus, regulating zinc homeostasis in the organelle (Ishihara et al., 2006). The Zn transporter protein 5 (ZNT5)-ZNT6 heterodimer in the early compartments of the secretory pathway promotes cellular sphingolipid metabolism by activating sphingosine phosphodiesterase 1 (Ueda et al., 2022). Moreover, levels of ZnT5 protein were significantly elevated in the brains of patients suffering from depression and suicide victims, indicating that ZnT5 could be involved in the pathophysiology of these conditions (Rafalo-Ulinska et al., 2016).

*ZnT6*

ZnT6 is expressed in the brain and liver. It often forms hetero-oligomers with ZnT5 and its immediate homologues in the early secretory pathways, which are responsible for loading zinc into zinc-deficient enzymes and maintaining the functions of these pathways (Fukunaka et al., 2009). This protein is localized to the Golgi and plays an important role in transporting cytoplasmic zinc to the Golgi and vesicular compartments (Amagai et al., 2023). In a study investigating the preclinical phase of AD, the levels of ZnT6 were found to be increased in the hippocampus and parahippocampal gyrus, while significantly decreased in the cerebellum of preclinical AD subjects compared to normal controls. This suggests that ZnT6 may be involved in the pre-pathophysiological changes occurring in AD (Lyubartseva et al., 2010). Currently, there is limited literature elaborating on the functions of ZnT6.

*ZnT10*

ZnT10 is expressed in the brain, the liver, prostate, and other organs, except in the pancreas. ZnT10 is mainly localized in the early or recirculating endosomal or Golgi membrane. However, ZnT10 can be expressed in the plasma membrane at high levels of extracellular zinc (Kambe et al., 2015). Additionally, ZnT10 plays an important role in zinc efflux, and the alteration in the expression levels of ZnT10 *in vivo* will affect homeostasis of manganese *in vivo* (Felber et al., 2019), potentially playing a role in the development of AD by affecting homeostasis of manganese *in vivo* (Bosomworth et al., 2013). Hara et al. (2017) found that individuals exhibiting ZnT10 mutations have a distinct phenotype of hypermanganism with dystonia, presented by neurological symptoms occurring in childhood (early onset) or adulthood (adult onset). In early-onset cases, children may present dystonia of the arms or legs, exhibit involuntary tremors, abnormally slow movements and experience slurred speech. However, those experiencing adult-onset include abnormal movements, tremors, abnormal slow movements, muscle tonus, and postural instability (Hara et al., 2017; Winslow et al., 2020). The ZnT3/ZnT10 heterodimer plays an important role in the transport of zinc into the organelle lumen by regulating epidermal growth factor receptor (EGF-R) signalling through increased phosphorylation of mitogen-activated protein kinase kinase (MEK) and extracellular signal-regulated kinase (ERK1/2) (Zhao et al., 2016). Decreased ZnT10 expression can play an important role in angiotensin II (Ang II)-induced senescence by increasing reactive oxygen species (ROS) and decreasing ERK1/2 phosphorylation-mediated down-regulation of catalase by post-transcriptional mechanisms (Patrushev et al., 2012).

#### Metallothioneins

MTs are a family of low molecular weight proteins rich in cysteine groups that are involved in various physiological and pathophysiological processes *in vivo*, which can play a pivotal role in the maintenance of zinc homeostasis by regulating oxidative stress (Baltaci et al., 2018). The differential expression of specific MT isoforms plays an important role in tumor growth, differentiation, angiogenesis, metastasis, microenvironmental remodeling, immune escape, and drug resistance and may serve as a novel target in diagnosis and treatment of tumors (Si and Lang, 2018). Studies elaborating on the function of MT4 are limited; therefore, the following sections will focus on MT1, MT2, and MT3.

*MT1*

MT1 is expressed in various organs and is not histologically specific. Zinc plays a key role in promoting the expression of MT1, which is involved in the formation of inflammation (Maybee et al., 2022). A study conducted by Yılmaz et al. (2023) showed that reduced concentrations of MT1 may lead to defective antioxidant defenses and an increased risk of schizophrenia. The activation of adrenergic β-receptors induces MT synthesis by increasing zinc levels in the body, thereby mitigating Aβ-induced neurodegenerative diseases (Kawano et al., 2022).

*MT2*

Like MT1, MT2 is expressed in most organs, with higher levels of MT2 occurring in the kidney, liver, intestines, and pancreas compared with other tissues. MT2 activates the expression of nuclear factor κB (NF-κB) and IκB-α promoters and plays an important role in ameliorating neuropathic pain induced by oxaliplatin (Huang et al., 2021). It has been shown that the melatonergic antidepressant agomelatine exhibits potent MT1 and MT2 melatonergic agonism, along with relatively weak antagonism of the 5-hydroxytryptamine 5HT2C receptor. Moreover, MT2 has been proven effective in the treatment of patients suffering from depression, marking MT2 as a promising therapeutic target for depression in the future (Feng et al., 2023).

*MT3*

MT3 is expressed in the brain, bone, and kidney, with particularly high levels in the brain. It may play a significant role in the development of AD as a growth inhibitory factor (Uchida et al., 1991). The upregulation of human MT3 increases resistance to cisplatin chemotherapy in neuroblastoma, suggesting that high levels of MT3 may indicate a poor prognosis for this condition (Rodrigo et al., 2021). The protein binding activity of MT3 derives from its β structural domain, while the α structural domain is not directly involved (Zaręba and Kepinska, 2020). Zaręba and Kepinska (2020) reported increased expression of MT1 and MT2, along with a 30% decrease in MT3 expression. However, results regarding increases or variations in MT3 expression in AD have not been established. Zaręba and Kepinska (2020) suggest that this may be related to the degree of AD progression, highlighting an existing research gap on this topic.

## Zinc Homeostasis Gene and Neurodegenerative Diseases

### Zinc homeostasis genes and neurological functions

Different zinc homeostasis genes are differentially expressed in various parts of the neuron, and their interconnections play an important role in maintaining neurological functions. The following section elaborates on the role of zinc homeostatic genes in neurons. A study conducted in 2015 found that ZnT1 is abundantly expressed in the NMDA receptor-enriched hippocampal postsynaptic density and specifically binds to the GluN2A subunit of the NMDA receptor, which is involved in excitatory synapse dynamics (Mellone et al., 2015). ZnT3 is an important presynaptic vesicle protein responsible for concentrating zinc ions in the synaptic vesicles of a subset of brain glutamatergic neurons, which is not observed with other zinc transport proteins. ZnT7 is expressed in the superior cervical ganglion in mice and may offer a neuromodulatory role in the adrenergic neuronal pathway in the mouse SCG (Gao et al., 2008). ZIP1 is expressed in several brain regions, such as the hippocampus, cerebellum, and thalamus. It is mainly located in the cell bodies of neurons, playing an important role in the uptake of endogenous zinc (Belloni-Olivi et al., 2009). ZIP3 is strongly expressed in hippocampal pyramidal neurons, helping to maintain zinc homeostasis (Qian et al., 2011). ZIP4 is expressed in neuronal dendrites and co-localizes with two scaffolding proteins of the hippocampal PSD, HOMER1 and SHANK3 (De Benedictis et al., 2021). In the preceding introduction, we have explored the relationship between zinc homeostasis and the maintenance of neurological function. In the following sections, we will describe the mechanisms by which disorders of zinc homeostasis are associated with neurological dysfunction.

### Molecular mechanisms linking zinc homeostasis and neurodegenerative disease

#### Oxidative stress

Previous studies have demonstrated that zinc can exert periodic antioxidant properties through four pathways: (1) Zinc can inhibit the production of hydroxyl radicals from redox-active metal-catalyzed H_2_O_2_ by competitively binding with redox-active metals, such as iron and copper, to proteins and cell membranes; (2) Zinc can bind to the sulfhydryl groups of biomolecules, protecting them from oxidation by oxidizing substances; (3) Zinc exerts its antioxidant properties by increasing the activity of antioxidant enzymes such as glutathione (GSH), catalase, and superoxide dismutase, while decreasing the activity of oxidation-promoting enzymes, including inducible nitric oxide synthase and nicotinamide adenine dinucleotide phosphate oxidase, and by inhibiting the production of lipid peroxidation products; (4) Zinc exerts remarkable antioxidant effects by inducing the expression of metallothionein, which has cysteine-rich, antioxidant properties (Cesak et al., 2023; Jia et al., 2025). Oxidative stress may be an important mechanism of neurodegeneration in AD (Mezzaroba et al., 2019). Mandal et al. (2023) found a depletion of brain GSH through magnetic resonance spectroscopy and quantitative drug sensitivity measurements in patients with AD. Another study on a transgenic mouse model of AD found that GSH depletion occurred before the deposition of amyloid plaques, thereby triggering the dimerization of cysteine disulfide bonds in tau proteins and leading to the subsequent formation of neurofibrillary tangles (Roy et al., 2023). These results support the notion that the formation of oxidative stress predominates in the onset of AD, indicating that neurotoxicity induced by oxidative stress is an important mechanism for the subsequent Aβ oligomerization and tau-mediated neurofibrillary tangles in AD. Imbalances in zinc homeostasis in AD and PD can trigger a cascade of reactions involving oxidative stress, structural disorganization, protein aggregation, mitochondrial dysfunction, and energy deficits, ultimately leading to disruptions in the normal functions of neuronal networks (Greenough et al., 2016). To investigate the role of zinc in maintaining redox homeostasis in the brain, Bagchi et al. (1998) administered mice a zinc-deficient diet (1^2+^2 mg/kg Zn for 5 weeks) and identified increased lipid peroxidation, DNA fragmentation, and ROS production in brain tissue, as well as depleted GSH levels. Administering 13 mg/kg Zn supplements for 4 days mitigated these health effects. Furthermore, impaired metabolism of GSH predisposes neurons to oxidative damage, consequently impairing neurological development (Wandt et al., 2021).

#### Neuroinflammation

**Figure 4:**
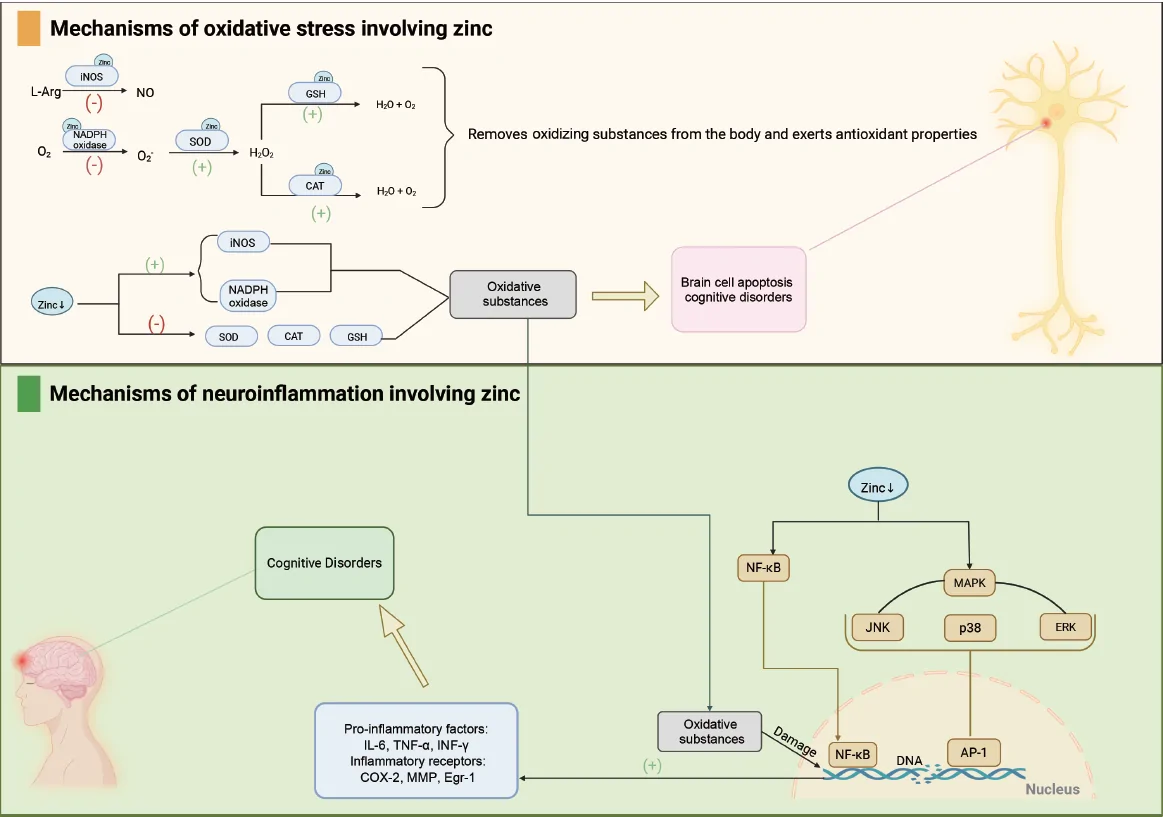
Zinc homeostasis and its role in oxidative stress and neuroinflammation in neurodegenerative diseases. In the mechanisms of oxidative stress, zinc can enhance the activity of antioxidant enzymes, including GSH, CAT, and SOD, by binding to their surface receptors. It can also inhibit the activities of oxidation-promoting enzymes, such as nitric oxide synthase and nicotinamide adenine dinucleotide phosphate oxidase, through receptor binding, ultimately exerting antioxidant effects in vivo. However, imbalances in zinc homeostasis reverse these effects, resulting in the accumulation of large amounts of oxidants in the body, which promote apoptosis of brain cells and contribute to neurodegenerative diseases. These oxidants are involved in neuroinflammatory mechanisms, leading to the overproduction of inflammatory mediators. In neuroinflammation, zinc prevents NF-κB from dissociating from its inhibitory protein, thereby preventing the translocation of NF-κB to the nucleus and subsequently inhibiting inflammation (Gammoh and Rink, 2017). When zinc levels decrease, NF-κB and AP-1 are activated via the NF-κB and MAPK/ERK pathways, increasing the production of pro-inflammatory cytokines and activating inflammatory receptors involved in the development of numerous neurodegenerative diseases (Sun et al., 2022). Created with BioRender.com. AP-1: Activator protein-1; CAT: catalase; COX-2: cyclooxygenase-2; Egr-1: early growth response-1; GSH: glutathione; IFN-γ: interferon gamma; IL-6: interleukin-6; MAPK/ERK: mitogen-activated protein kinase/extracellular signal-regulated kinase; MMP: matrix metalloproteinase; NF-κB: nuclear factor-κB; SOD: superoxide dismutase.

Zinc serves as an anti-inflammatory agent in most inflammatory signaling pathways (Chai and Cao, 2023; Islam et al., 2023). For example, low levels of zinc in inflammatory diseases, including COVID-19 and sepsis, elevate the levels of inflammatory mediators such as interleukin-16 (IL-6) and tumor necrosis factor-alpha (TNF-α), necessitating zinc supplementation (Briassoulis et al., 2023). Similarly, zinc prevents nuclear factor kappa B (NF-κB) dissociation from its associated inhibitory proteins, thereby inhibiting the nuclear translocation of NF-κB and subsequently reducing inflammation. Additionally, zinc inhibits IL-6-mediated activation of STAT3, contributing to its anti-inflammatory effects (Gammoh and Rink, 2017). Furthermore, a study conducted among older adults revealed that impaired zinc homeostasis worsens immune dysfunction and chronic inflammation, which are the primary pathophysiological changes that occur during the aging process (Giacconi et al., 2017). Moreover, zinc deficiency may contribute to neuroinflammation by altering normal gut function and the composition of gut microbiota, which is a crucial mechanism involved in the onset of autism in mice (Sauer et al., 2021). The overexpression of Zinc-α2-glycoprotein, an anti-inflammatory adipocytokine, may suppress the occurrence of epileptic seizures by inhibiting the phosphorylation of ERK mediated by transforming growth factor beta, TNF-α, and IL-6-mediated neuroinflammation (Liu et al., 2018). Early growth response-1 is a zinc-finger transcription factor whose early activation worsens neuroinflammation and dopaminergic neurodegeneration, contributing to the pathophysiological changes associated with PD (Yu et al., 2018). Similarly, a study investigating the relationship between neuroinflammation and the repair of cortical tissues found that the activation of pro-inflammatory factors reduced the levels of microglia by upregulating zinc-dependent integrins and metalloproteinase 17, which consequently led to the cleavage of the CSF-1 receptor (CSF-1R), halting neuroglial repair (Hernandez-Espinosa et al., 2025). Inflammation plays a decisive role in zinc-induced dopaminergic neurodegeneration in the substantia nigra striata. COX-2 causes endogenous apoptosis in PD, which is induced by zinc through the activation of microglia, triggering the release of inflammatory mediators and leading to dopaminergic neurodegeneration (Chauhan et al., 2018; **Figure 4**).

#### Zinc-mediated neuronal cell death

Neurological function depends on the normal functioning of neurons, and neuronal cell death mediated by zinc is one of the key mechanisms by which impaired neuronal function leads to neurological deficits. Currently, zinc oxide nanoparticles (ZnONPs) are vital nanomaterials widely used in various applications. It has been found that exposure to ZnONPs induces the activation of c-Jun N-terminal kinase in neuronal cells in the cerebral cortex of mice, producing an iron death phenotype (Qin et al., 2020). Tuo et al. (2018) discovered the essential role of zinc in the activation of cyclin-dependent kinase 5 (CDK5) during cerebral ischemic injury. Additionally, the phosphorylation of CDK5 at Tyr15 induced by zinc promotes the activation of CDK5, ultimately resulting in ischemic neuronal death in patients suffering from stroke. Previous studies have suggested that zinc-induced cell death may play an important role in the pathogenesis of PD (Granzotto et al., 2020; Pradhan et al., 2023). By comparing mice fed zinc and dopamine with normal mice, Yang et al. (2016) found that the expression of Gadd45b protein and the levels of p38 phosphorylation were increased in the substantia nigra of the experimental group. Enhanced expression of Gadd45b protein accelerated apoptosis in PC12 cells, a process closely related to the pathogenesis of PD. ZnT86D, a direct homologue of ZnT7, may play a key role as a protective factor in neurodevelopment and the pathogenesis of AD. Silencing its expression results in impaired neurogenesis and neuronal cell death (Lee et al., 2022). A previous study showed that excessive zinc influx triggers the overactivation of protein kinase C (PKC), Src, extracellular signal-regulated kinase (ERK), as well as NADPH oxidase and poly (ADP-ribose) polymerase, ultimately leading to neuronal death. This could represent a molecular link between zinc imbalance and neurodegenerative diseases (Eom et al., 2024). The molecular mechanisms associated with zinc-mediated ferroptosis in the development of neurodegenerative diseases are still relatively limited, requiring further exploration in future research.

In this section, we described the mechanisms of zinc homeostasis imbalance and its effects on neurological dysfunction, establishing the molecular foundation for understanding the altered expressions of zinc homeostasis genes and the development of neurodegenerative diseases. The role of homeostasis genes in different neurodegenerative diseases is explained below.

### Role of zinc homeostasis genes in neurodegenerative disease

In recent years, a large number of original studies have confirmed that abnormalities in zinc and zinc homeostasis genes *in vivo* are involved in the development of a variety of neurodegenerative pathologies (Zhong et al., 2024; Chen et al., 2025).

The following sections describe the alterations in zinc homeostasis genes in AD, PD, and schizophrenia. The first part elaborates alterations occurring in zinc homeostasis genes AD.

#### Alzheimer’s disease

AD is one of the most common neurodegenerative pathologies characterized by the accumulation of two proteins, Aβ and tau (associated with microtubules), which lead to the formation of amyloid plaques and neurofibrillary tangles, respectively (Kim et al., 2025; Yao et al., 2025; Zhang et al., 2025). These changes are thought to contribute to the development and progression of AD (Jin et al., 2025; Long et al., 2025; Zhao et al., 2025). Although the exact relationship between these two pathologies is currently unknown, evidence suggests that they may interact in a bidirectional manner to promote their aggregation and increase toxicity (Jomova et al., 2022; Roda et al., 2022). Recent research has focused on the hyperphosphorylation of tau proteins and the dysregulation of Zn^2+^ homeostasis. Some studies have demonstrated that zinc could be involved in the pathogenesis of AD by interacting with Aβ, APP, and Zn^2+^-mediated tau aggregation and neurotoxicity. For instance, Wang et al. (2010) found that in APP and PS1 double transgenic mice, increased expression of APP enhanced amyloid APP cleavage and Aβ deposition, impairing spatial learning and memory in mice treated with a high dose of zinc (20 mg/mL zinc sulfate in drinking water) (Wang et al., 2010). Additionally, Li et al. (2019) examined the effects of multiple metal ions on tau proteins and discovered that Zn^2+^ produced oligomers when incubating tau-R3 plaques with transition metal cations. Conversely, the morphology of tau-R3 was not altered in the presence of other metal cations. The formation of tau-R3 oligomers is a primary cause of neurotoxicity; the induction of ROS by Zn^2+^ exacerbates the effects of tau-R3 on the toxicity of peripheral neuronal cells, which was investigated through cell counting in flow cytometry and by detecting ROS produced by the cells using 2,7-dichlorodihydrofluorescein diacetate. Overproduction of ROS leads to mitochondrial dysfunction and oxidative stress, followed by neuronal death (Li et al., 2019). AD can be divided into four stages: preclinical AD, mild cognitive impairment, early AD, and late AD (Tramutola et al., 2015). In contrast to patients with preclinical AD, who exhibit low expression of ZnT1 and elevated expression of ZnT6 in the hippocampus, patients with early and late AD show elevated levels of ZnT4 and ZnT6, while the expression levels of ZnT1 are increased in the hippocampal gyrus (Smith et al., 2006). These alterations in the expression of ZnT1 may be related to increased concentrations of extracellular zinc, which exacerbate the deposition of Aβ (Xu et al., 2019).

Some researchers have found that levels of ZnT3 are considerably lower in patients with AD, possibly due to insufficient release of zinc from synaptic vesicles into the synaptic gap, resulting in impaired inhibition of zinc at postsynaptic receptors and neurological zinc deficits (Beyer et al., 2009; Adlard et al., 2010). Moreover, levels of ZnT10 mRNA are reduced in the frontal cortex of patients with AD; however, the underlying cause remains unclear (Bosomworth et al., 2013). ZnT7 may play an important role as a potential protective factor in the development of AD (Lee et al., 2022). MTs are also involved in the development of AD. Substantial evidence shows that expression levels of MT-1/2 are increased, while that of MT-3 is decreased in the brains of patients with AD, consequently affecting their function (Zaręba and Kepinska, 2020). MT-2 decreases the ability of transthyretin to bind to Aβ and promotes Aβ aggregation by interacting with TTR, unlike MT-3, which increases TTR-Aβ binding and inhibits Aβ aggregation (Zaręba and Kepinska, 2020), consistent with the phenomenon indicating that Aβ aggregation is a pathogenic mechanism in AD. MT-3 is exclusively expressed in the CNS and is considerably downregulated in AD (Squitti et al., 2020). Recent studies have demonstrated that this protein protects cultured neurons from the toxicity generated by Aβ. Additionally, the exchange of Zn^2+^ metal between MT-3 and soluble aggregates of Aβ_1–40_-Cu inhibits the production of reactive oxidants and the resultant cytotoxicity, potentially serving as a target for the development of AD therapies in the future (Squitti et al., 2020). Oxidative stress induced by aberrant homeostasis of transition metals may be important in the development of AD, and in the nervous system, metal-protein interactions appear to be important variables in the development or inhibition of neurodegeneration. Therefore, it has been suggested that metal chelation therapy may be an option for the treatment of transition metal ion homeostasis dysregulation-induced neurodegeneration (Singh et al., 2024).

#### Parkinson’s disease

PD is a common neurodegenerative condition characterized by delayed movement, resting tremor, muscle tonus, and decreased involuntary movements. Additionally, PD manifests various non-motor symptoms, including psychiatric symptoms and neurological impairments (Kalia and Lang, 2015). Currently identified mechanisms of PD include α-synuclein aggregation, oxidative stress, ferroptosis, mitochondrial dysfunction, neuroinflammation, and intestinal flora dysregulation. The interplay among these molecular mechanisms makes the pathogenesis of PD extremely complex (Dong-Chen et al., 2023). The pathological manifestation of PD involves the loss of dopaminergic neurons within the substantia nigra compacta, which subsequently leads to reduced dopamine secretion. The role of zinc in this pathological change has been investigated in previous studies (Jomova et al., 2022; Mizuno et al., 2024). For example, Tamano et al. (2019) found that increased levels of toxic Zn^2+^ in the cytoplasm may result from the influx of extracellular zinc into dopaminergic neurons, with the involvement of ZTPNs. Additionally, the combined infusion of intracellular Zn^2+^ chelators, such as ZnAF-2DA and tetrakis-(2-pyridylmethyl)ethylenediamine (TPEN), alongside 6-hydroxydopamine or paraquat into the substantia nigra, reduced the loss of dopaminergic neurons and associated motor deficits in the substantia nigra striata of rats. This suggests that the levels of Zn in the intracellular space have bifacial effects in PD (Tamano et al., 2018). Therefore, zinc treatment may exacerbate the progression of PD by activating NADPH oxidase, depleting GSH, increasing oxidative stress, impairing cellular function, and triggering apoptosis, which leads to neuronal loss (Kumar et al., 2012).

In conclusion, zinc influences PD in two different ways: (1) it prevents oxidative stress by affecting the activity of antioxidant enzymes and signaling pathways; and (2) it exerts deleterious effects due to the generation of excess Zn^2+^ in the oxidative stress pathways induced by intracellular Zn^2+^. In addition to alterations in the intracellular Zn pool, emerging evidence suggests that dysfunctional signaling of synaptic Zn^2+^ may also contribute to the pathogenesis of PD (Sikora and Ouagazzal, 2021). However, specific zinc homeostasis genes are still poorly investigated, necessitating further research to support this phenomenon. Levodopa-modified zinc oxide nanoparticles could be effective in preventing and treating PD by increasing the activity of glutamate transporter proteins, thereby enhancing neuronal cell viability and improving behavioral performance (Yeni et al., 2024).

#### Schizophrenia

Schizophrenia is characterized by psychotic symptoms, and cognitive impairment is now considered an additional clinical feature of the disorder (Javitt, 2023). Enlarged lateral ventricles and reduced brain volume are currently identified pathological changes in schizophrenia, the pathogenesis of which may be related to neurochemical disturbances in dopamine function and glutamatergic NMDA receptor function (Jauhar et al., 2022). Elevation of ZIP12 in the Brodmann region may promote a breakdown of zinc homeostasis in the CNS and be involved in the pathophysiology of schizophrenia (Dean et al., 2024). The mechanism of action may involve elevated ZIP12 expression remarkably reducing muscarinic M1 receptor mRNA expression in the cortex, which is implicated in the pathological process of muscarinic receptor-deficient schizophrenia (Zhang et al., 2024).

Some researchers have found a strong association between decreased expression of ZnT3 and schizophrenia in European samples, particularly among female participants (Perez-Becerril et al., 2016). This phenomenon was confirmed in a recent study, which demonstrated that the presence of two minor alleles, rs11126936 and rs11126929, generated by a variant in the ZnT3 gene, could be mediated by a reduction in dorsolateral anterior cingulate activity. Cortical glutamate plays an important role in the pathogenesis of schizophrenia (Davis et al., 2021). In a study of 48 ethnically diverse psychiatric cases, Kranz et al. (2016) found that decreased expression of ZIP13 resulted in severe neurological deficits and poor educational attainment, suggesting that variation in ZIP13 increases susceptibility to schizophrenia. However, substantial findings have shown no correlation between ZnT8 and ZnT4 and schizophrenia (Küry et al., 2003; Zhang et al., 2012).

### Therapeutic potential of zinc homeostasis genes in neurodegenerative diseases

Zinc plays an important role in the development of neurodegenerative diseases. Alterations in zinc levels can lead to degenerative diseases through various pathways, such as oxidative stress, which causes nerve cell death. Additionally, zinc may be used to reverse neurodegenerative diseases, making zinc supplementation a potential therapeutic target. A study conducted on patients with AD demonstrated the effectiveness of zinc in reducing levels of non-cyanine copper and showed potential to improve neurological performance without remarkable side effects (Squitti et al., 2020). Moreover, in a study of AD animal models, zinc deficiency led to increased NLRP3-driven inflammation, exacerbating neurological decline, which could be ameliorated by zinc supplements (Rivers-Auty et al., 2021). Patients with ASD and Phelan-McDermid syndrome are often zinc deficient, and zinc supplementation may reduce behavioral deficits in individuals suffering from these conditions (Alsufiani et al., 2022). The combination of oxiracetam and zinc may exhibit superior anti-autistic effects through various mechanisms, such as antioxidant, anti-inflammatory, and anti-excitotoxic actions, providing novel ideas for clinical treatment options for ASD (Paudel et al., 2020). A systematic evaluation and meta-analysis examining the effects of zinc supplementation in combination with antidepressant medications in depressed patients suggest that zinc supplementation may reduce depressive symptoms in individuals receiving antidepressant treatment (da Silva et al., 2021). Additionally, a study of AD in a Drosophila model, which targeted the knockdown of ZIP1, revealed improved AD symptoms and prolonged survival time (Lang et al., 2012). ZnT3 knockout mice exhibit learning and memory deficits (Adlard et al., 2010), while ZnT7 may act as a potential protective factor in the onset of AD (Lee et al., 2022). Research has shown that ZnT3-deficient mice may exhibit autism through mechanisms such as an imbalance of zinc homeostasis, activation of MMP-9, and the upregulation of brain-derived neurotrophic factor (Yoo et al., 2016). The production of MT-1/2 by astrocytes in response to oxidative stress offers protective effects against damage to dopaminergic neurons; targeting MT in astrocytes emerges as a novel approach for PD treatment (Miyazaki and Asanuma, 2021).

Blood–brain barrier permeability is a key indicator for assessing the applicability of drugs in the treatment of neurodegenerative diseases in clinical settings. The octanol/water partition coefficient (Pow) is widely used in bioactivity prediction studies. For example, Behar and Maayan (2024) measured the octanol/water partition coefficients of two peptide-based zinc chelators (APD2 and PT1), revealing log Pow values of 1.05 for APD2 and 0.52 for PT1, both within the predictive range of 0.3 to 3. This suggests that these peptide-based zinc chelators are likely to cross the blood–brain barrier, demonstrating therapeutic potential. Xie et al. (2021) used the parallel artificial membrane permeation test method to measure lipid extracts of porcine brain and investigated the blood-brain barrier permeability of 11-substituted sampanginin derivatives of a novel zinc chelator. The results showed that all 11-substituted sampanginin derivatives had permeability values higher than 222, indicating that these molecules could cross the blood-brain barrier by passive diffusion.

### Safety

Adverse reactions and cytotoxicity may occur during zinc homeostasis gene-targeted therapy, and zinc therapy may unavoidably lead to excess zinc-mediated cytotoxicity. Therefore, the safety of zinc imbalance therapy is a critical consideration, and this section describes the possible adverse reactions associated with zinc imbalance therapy.

Zinc therapy for neurodegenerative diseases is primarily used in Wilson’s disease, with a recommended dose of 50 mg elemental zinc tablets (2–3 tablets per day) considered appropriate for Wilson’s disease therapy. However, this dosage may result in adverse effects in individuals with mild cognitive impairment who exhibit values above the non-copper blue values, such as iron granulocytic anemia (uncommon) and elevated levels of serum amylase, lipase, and alkaline phosphatase (common) (Squitti et al., 2020). The most common adverse reactions caused by zinc sulfate include gastritis (stomach pain) and elevated pancreatic enzyme levels (without clinical signs) (Członkowska et al., 2014). A report on zinc sulfate treatment in patients with Wilson’s disease noted that among 72 patients who initiated treatment with zinc sulfate, only 2 (2.7%) required a change in treatment due to adverse reactions, which included skin reactions and thrombocytopenia (Członkowska et al., 2014). The first sign of zinc overtreatment is usually mild anemia, with or without leukopenia, which can be reversed by lowering the zinc dose or, in more severe cases, temporarily discontinuing the drug (Litwin et al., 2023). In early clinical trials of zinc-based therapies, it has been suggested that during the initial phase, copper blue protein levels and blood counts should be assessed at 3, 6, and 12 weeks for zinc overtreatment. If hemoglobin, white blood cell counts, or platelets decrease by 20% or more, or if the cuprocyanin value is less than 10 mg/dL, zinc administration should be temporarily suspended until counts recover and then resumed at 50% of the previously administered dose (Squitti et al., 2020). If a relapse occurs, discontinuation is recommended until neither mild anemia nor leukopenia is present, as assessed by blood counts, with a minimum dose of one elemental zinc tablet (50 mg/day) (Squitti et al., 2020).

The use of zinc chelators for the treatment of AD causes meningoencephalitis as an adverse effect in approximately 6% of patients, although the exact mechanism remains unclear (Nordberg, 2003). In a randomized, double-blind trial involving 36 patients with AD treated with chloroquine (a zinc chelator), one subject in the treatment group developed neurological symptoms (impaired visual acuity and chromatic sensation), which subsided after discontinuation of treatment, possibly as a result of the drug (Sampson et al., 2008). An 8-hydroxyquinoline derivative, a synthetic zinc chelator, was investigated for cytotoxicity against a human glioma cell line (T67) and primary human umbilical vein endothelial cells at concentrations ranging from 0.5 to 50 mM. It was found that cell viability was maintained at 91% of the original level even at the highest experimental concentration (Prati et al., 2016). In conclusion, while the safety of zinc chelator therapy appears to be assured, the reasons for the occurrence of adverse effects and the mechanisms by which they arise need to be elucidated in more in-depth studies. As nanoparticles and nanofibers are increasingly produced and used industrially, the development of functionalized nanoparticles for medical therapeutic applications is gradually rising.

## Current Status of Clinical Translation and Application

Conducting clinical trials is often more challenging than performing animal studies. As of August 21, 2025, there were six registered research projects on zinc therapy for neurodegenerative diseases at the North American Clinical Trials Registry Center. This review omits unregistered studies. In a study on zinc therapy for amyotrophic lateral sclerosis (NCT01259050), patients exposed to high levels of zinc exhibited reduced excitotoxicity from free glutamate and glutamate toxicity, leading to symptom relief without adverse events. This study strongly supports the application of zinc therapy in amyotrophic lateral sclerosis. Additionally, a long-term study on the efficacy and safety of zinc acetate for Wilson’s disease (NCT00212355) reported that the incidence of adverse events over a 10-year follow-up period was only 5%, with no severe adverse events occurring. Therefore, relevant clinical studies have confirmed the feasibility of zinc therapy in certain neurodegenerative diseases. **Additional Table 3** summarizes the project information currently registered at ClinicalTrials.gov. **Additional Table 3** helps researchers understand the trends and current status of zinc in clinical treatment studies for neurodegenerative diseases. However, such clinical trials are currently limited to Wilson’s disease and amyotrophic lateral sclerosis, and registered studies on zinc therapy for most neurodegenerative diseases remain scarce. Additionally, results from some registered projects have yet to be published. Regarding the application of zinc therapy in neurodegenerative diseases, most studies are still in the preclinical animal experiment stage. Further clinical trials are needed to validate the safety of zinc therapy in neurodegenerative diseases.

**Additional Table 3:** Information on ongoing and completed clinical studies at ClinicalTrials.gov includes details on intervention measures, routes of administration, dosages, and other project specifics

NCT number	Title	Country	Clinical trial phase	Zinc treatment strategies			
				Interventions	Route of administration	Dosage	Population
NCT00212355	Efficacy and safety, long-term study of zinc acetate to treat Wilson's disease in Japan	Japan	Phase 3	Zinc acetate	Oral	For patients aged 1-5 yr, 25 mg twice daily; for patients aged 6 to 15 yr, 25 mg three times daily; for patients aged 16 yr and older, 50 mg three times daily.	Enrollment: *n* = 37; age: ≥ 1 yr; sex: men and women
NCT00004338	Study of zinc for Wilson disease	United States	Phase 4	Zinc acetate	Oral		Enrollment: *n* = 300; age: ≥ 0 yr sex: men and women
NCT04983667	Zinc-AA supplementation during pregnancy & lactation to assess effects on ASD prevalence in offspring	Mexico		Zinc dietary supplements	Oral	30 mg/d	Enrollment: *n* = 109; age: 18-35 yr; sex: women
NCT00325572	Evaluation and treatment of copper/zinc imbalance in children with autism	United States	Phase 1	Vitamin C supplements Zinc	Oral		Enrollment: *n* = 89; age: 3-8 yr; sex: men and women
NCT01259050	Safety study of high doses of zinc in ALS patients	United States	Phase 1 Phase 2	Copper Optizinc	Oral	Optizinc 90 mg/d; copper 1 mg/d	Enrollment: *n* = 10; age: 18-85 yr; sex: men and women
NCT03659331	A controlled study of potential therapeutic effect of oral zinc in manifesting carriers of Wilson disease	Israel		Zinc Dietary Supplements	Oral	300 mg/d	Enrollment: *n* = 50; age: ≥ 18 yr; sex: men and women

The stage of a clinical trial studying a drug or biological product is defined by the U.S. Food and Drug Administration. The phase is determined by the study's objective, the number of participants, and other characteristics. There are five phases: early phase 1 (formerly listed as phase 0), phase 1, phase 2, phase 3, and phase 4. Projects not specified in these stages do not apply to this classification. The information provided in this table is sourced from data collected from ClinicalTrials.gov on August 21, 2025.

## Limitations

This review is based on literature that effectively highlights knowledge gaps in zinc homeostasis and nerve damage. Notably, the primary knowledge gap pertains to the specific structure of the ZIPs, although these findings require further investigation. The roles of ZIP11, ZnT6, and ZnT9 in organisms and their relationship to disease have not been fully characterized. It is hoped that more research will be devoted to elucidating these relationships in the future. In terms of mechanisms of action, the phenomenon of zinc imbalance leading to synaptic dysfunction has not been fully elucidated. The molecular mechanisms underlying zinc homeostasis-mediated synaptic dysfunction and neuronal cell death are currently unclear, and studies of related downstream signaling pathways are insufficient. From a therapeutic perspective, studies on zinc homeostatic genes as clinical gene therapy targets are adequate for AD and tumor cells, but there are knowledge gaps in PD, ASD, schizophrenia, HD, and ALS. Currently, there are limited translational studies on zinc homeostasis genes, and most of them focus on tumor therapy. It is hoped that an increasing number of translational studies will focus on zinc homeostasis genes in neurodegenerative diseases in the future. Regarding the safety of zinc therapy, the use of zinc in treating neurodegenerative diseases has yet to be determined. While most studies focus on animal research, clinical trials are insufficient. Systematic assessments and meta-analyses of drug safety in large sample sizes are still lacking, emphasizing the need for further research on the clinical safety of zinc therapy.

## Future Perspectives and Conclusions

In recent years, researchers have mainly proposed the following therapeutic approaches for the treatment of neurodegenerative diseases: targeted synaptic function and deletion therapy, stem cell–based cell therapy, targeted iron death, hyperbaric oxygen therapy, and gene therapy with viral vectors (Dejanovic et al., 2024). This paper proposes zinc homeostasis gene as a novel therapeutic target for neurodegenerative diseases. This target is likely to serve as an important reference for future neurodegenerative disease therapies.

Alterations in zinc homeostasis *in vivo* affect the expression of zinc transporter proteins. Abdo et al. (2021) detected the expression levels of zinc transporter proteins in endothelial and smooth muscle cells under different zinc conditions using fluorescence confocal microscopy. They concluded that the mRNA expression of ZnT1, ZnT2, and MT1 was significantly downregulated under low and high zinc conditions, whereas the expression of ZIP2 and ZIP12 was induced by zinc depletion with the zinc chelating agent TPEN (Abdo et al., 2021). Experimental studies have demonstrated the efficacy of zinc chelators in the treatment of neurodegenerative diseases, such as AD and PD (Matsuyama et al., 2021; Teil et al., 2022). The deposition of Aβ protein in AD is thought to be a significant factor in the disease’s pathogenesis (Guo et al., 2020). TPEN, a specific zinc chelator, attenuates Aβ25-35-induced neurotoxicity. The results of Chen et al. (2023) revealed that the potential mechanism of TPEN is its ability to reduce Aβ_25–35_-induced neuronal death by lowering intracellular Zn^2+^ and Ca^2+^ concentrations. This, in turn, reversed the Aβ_25–35_-induced increase in ROS content and reduced Aβ_25–35_-induced neuronal damage (Chen et al., 2023). This review confirms the feasibility of zinc chelation as a treatment for neurodegenerative diseases.

Currently, small molecular peptides have become a hotspot in zinc chelator research. A casein-derived peptide (TEDELQDKIHP) was identified to chelate zinc, forming TEDELQDKIHP-Zn complexes, which are well-absorbed bioparticles that promote trans-epithelial transport of zinc (Li et al., 2023). Recently, a new design structure for silicon-based zinc ion carriers has been proposed by Yamada et al. (2022), yielding ion carriers with better performance than many current Zn^2+^ carriers and higher zinc specificity. This holds significant reference value for the development of new chelating agents in the future. With increasing breakthroughs in biopharmaceutical technology, more potent zinc chelators have been discovered and produced. However, there is a lack of research on novel zinc compounds in neurodegenerative diseases. Such drugs may be a hidden treasure for the treatment of neurodegenerative diseases.

Measurement of zinc levels in the body can be quantified using laser ablation-inductively coupled plasma mass spectrometry and solution atomization-inductively coupled plasma mass spectrometry (Hosseinpour Mashkani et al., 2024). Additionally, it has been suggested that zinc levels in the brain can be quantitatively determined using a combination of a flame atomic absorption spectrophotometer and a hollow cathode lamp (Haşimoğlu et al., 2022). The ability to measure zinc levels in the brain reinforces the feasibility of zinc homeostasis genes as therapeutic targets, and monitoring changes in zinc levels in the brain is crucial for determining medication dosage and preventing adverse effects during zinc therapy. Kim et al. (2021) illustrated the relationship between serum zinc levels and the deposition of Aβ in the brain of patients with AD. The fact that serum zinc levels are relatively easy to measure indicates significant potential for zinc homeostasis gene-targeted therapy. If future studies can clarify the relationship between quantitative measurements of serum zinc levels and neurodegenerative diseases, zinc homeostasis gene-targeted therapy may become a highly feasible clinical treatment option for these conditions.

In this review we reviewed the considerable role of zinc as a micronutrient and described the process of zinc uptake *in vivo*, as well as the link between zinc and neurological functions, establishing a strong foundation for connecting zinc homeostasis genes to neurodegenerative diseases. Additionally, we presented the distribution and major roles of zinc homeostasis genes *in vivo*, suggesting the effects of their altered expressions associated with various diseases, particularly their important role in neurodegenerative diseases. We also discussed the molecular mechanisms linking zinc homeostasis genes to neurodegenerative diseases and illustrated the potential of zinc homeostasis genes as therapeutic targets, providing novel insights for the treatment of neurodegenerative diseases such as AD in clinical settings. Increasing zinc levels through exogenous zinc supplementation has been reported to improve the prognosis of several neurodegenerative diseases. For example, exogenous zinc supplementation has proven effective in reducing non-copper levels and improving neurological performance in patients with AD, who exhibited no considerable side effects (Squitti et al., 2020). Exogenous zinc supplementation can also improve behavioral deficits in patients with ASD and pediatric movement disorders (Alsufiani et al., 2022). This reinforces the importance of zinc homeostasis genes as potential therapeutic targets for neurodegenerative diseases. However, there are considerable gaps in research regarding the clinical translation and application of zinc therapy, emphasizing that future studies should primarily focus on this area.

## Additional files:

***Additional Table 1:*** *Functions and localization of the ZIP family.*

***Additional Table 2:*** *Functions and localizations of the ZnT family.*

***Additional Table 3:*** *Information on ongoing and completed clinical studies at ClinicalTrials.gov includes details on intervention measures, routes of administration, dosages, and other project specifics.*

## Data Availability

*All relevant data are within the paper and its Additional files.*
